# Diversity, Inclusive Leadership, and Health Outcomes

**DOI:** 10.15171/ijhpm.2020.12

**Published:** 2020-01-26

**Authors:** Elizabeth H. Bradley

**Affiliations:** Vassar College, Poughkeepsie, NY, USA.

**Keywords:** Diversity, Inclusive Leadership, Health Outcomes, Health Policy and Management

## Abstract

In this time of polarization and divisiveness across increasingly diverse communities, health policy and management research offers an important insight: engaging diversity meaningfully through inclusive leadership—that embraces staff across hierarchies and engages difference perspectives so that all healthcare workers of all kinds feel they can speak up and participate—can save lives. In multiple studies of quality in cardiovascular care, top performing hospitals have been shown to exhibit the capacity to embrace staff across hierarchies and engage differences so that healthcare workers of all kinds feel they can speak up and participate meaningfully in improvement efforts. Most recently, in the two-year, longitudinal Leadership Saves Lives study of 10 hospitals, the ability to adopt a culture of improvement rather than blaming was linked to significant reductions in risk-standardized mortality rates. Moreover, the guiding coalitions (ie, quality improvement teams) in six of the 10 hospitals that were most successful were distinguished in three ways that give insight about effective modes of engaging differences: (1) including staff from difference disciplines and levels in the organizational hierarchy, (2) encouraging authentic participation by the members, and (3) using constructive patterns of managing conflict (ie, having clear role definitions, working to surface minority viewpoints, and collectively revisiting the shared goal of saving lives). Based on this literature, adequately engaging a wide range of diverse viewpoints and staff roles can have a marked impact on health outcomes. Although the studies reviewed do not examine racial/ethnic diversity per se, they do lend insight into effectively navigating environments with extensive diversity of perspectives, professional identities, and experiences. Future research may assess whether these insights have application to other forms of diversity as well. In this time of extreme polarization and division globally and locally, health policy and management research has an opportunity to share evidence that could help navigate an increasingly diverse environment, at least within the field of healthcare, towards a more inclusive, humane, and life-giving approach to our collective future.

Evidence of polarization and divisiveness appears regularly in the headlines in this moment in history. The movement toward division and unrest is not uniquely American but rather seems to be sweeping the globe—from Hong Kong to Haiti and many populations in between. Although these large social movements lie beyond the realm of health policy and management research, a key insight from our field is that engaging differences in perspectives and roles through an inclusive, rather than a divisive, approach to leadership can improve health outcomes, across an array of metrics. Given the current social and political context, health policy and management research has an important role to play in demonstrating the value of inclusive leadership for health of patients, families, and the larger community.

## First, the Facts

Our global interconnectedness and resulting diversity have increased enormously in the last fifty years.^1^ Not only has the global population doubled in the last fifty years, but also people are crossing international borders more frequently than ever, with 1.4 billion tourist arrivals globally in 2014.^2^ Additionally, more than one billion people–nearly 15% of the global population–are migrant, with about 30% of them relocating internationally and 70% moving within countries.^3^ In the United States, nearly 15% of the population is of immigrant status, up from a low of 5% about 50 years ago.^4^ The last time the immigrant population was this large (the 1920s), US immigrants were largely English speaking and of European descent, and nonetheless, a surge in Ku Klux Klan activity resulted. Today, the largest portions of our immigrant populations are Mexican, Indian, and Chinese—with language and cultural differences that enrich and challenge the status quo. In short, with the population becoming increasingly heterogeneous, the challenges and opportunities to engage differences in viewpoints and experience are omnipresent.

## Second, the Opportunity

Within the healthcare sector, diversity in perspectives, experience, and training among healthcare workers abounds, which can enrich the breadth of understanding of effective and appropriate care for diverse populations. The US healthcare workforce is blessed with foreign-born workers, who comprise 25% of physicians and registered nurses and 20% of other direct care workers in the United States.^5^ Although many decry the extra time and commitment required to manage a diverse workforce, to inspire a team of people who do not see the world the same way, or to simply navigate colleagues whose perspectives may offend—the important question is not *whether* to engage but *how* to engage.

Two schools of thought have emerged around the challenges of diversity. One school of thought is articulated by Professor Scott Page from the University of Michigan in his text, *The Difference* .^6^ In the first, Page argues that “diversity trumps ability” under certain conditions. These conditions include (1) the problem is difficult, (2) the people involved have good backgrounds for addressing the problem, (3) the people are able to improve upon each other’s first attempts to solve the problem, and (4) the people are drawn from a large population of potential problem solvers. Through a series of mathematical and analytic steps, Page then supports his assertion that groups that are diverse will more likely find the right solution than groups that are homogeneous. In contrast, a second school of thought is described by authors such as political commentator Heather MacDonald in *The Diversity Delusion*^7^ and Professor Anthony Kronman from Yale University, in *The Crumbling of American Excellence* .^8^ These authors fear that diversity and excellence are in competition, and favoring diversity too often results in sacrificing excellence in performance.

What seems to be missing in both schools of thought is the key understanding that presence of diversity alone does not predict performance; rather how diversity is *engaged* is central to whether diversity will improve or inhibit group performance. A common adaptation to diversity (and its inherent conflict) is what organizational analyst and journalist William Whyte, author of *The Organizational Man,* called “groupthink”^9^ wherein diversity of opinion is squelched by social pressure, a phenomenon Professor Irving Janis from Yale University demonstrated led to blind spots and poor group decision-making.^10^ Today, researchers such as Professor Amy Edmondson at the Harvard Business School, Professor Ingrid Nembhard at the University of Pennsylvania Wharton School of Business, Professor David Berg at the Yale Medical School, and Professor William Kahn at the Boston University School of Management are each writing and researching about concepts of psychological safety,^11^ inclusive leadership,^12^ and managing paradox in groups.^13,14^ These contemporary thinkers collectively have produced ample research on the importance of not only attracting but also fully engaging diverse groups so that differences of opinions may surface, be openly discussed without fear of mocking or ostracization, and so that the inevitable conflicts may be channeled into productive innovation and work.

## Third, Empirical Evidence in Healthcare

Complementing the plethora of theoretical literature on this topic of diversity is a growing empirical literature that reveals patterns related to engaging diversity successfully within healthcare organizations. The capacity to engage different voices and channel potential dissonance into creative problem solving and collaboration has been described as at the heart of learning and inclusive leadership.^15,16^ In several studies of quality in cardiovascular care, from beta-blocker use,^17^ to door-to-balloon time,^18^ to in-hospital cardiac arrest resuscitation,^19^ to risk-standardized mortality,^20^ top performing hospitals have been shown to exhibit the capacity to embrace staff across hierarchies and engage differences so that healthcare workers of all kinds feel they can speak up and participate. In these studies, having a shared goal to re-focus and align people in the face of conflict has been critical. Most recently, in the two-year, longitudinal Leadership Saves Lives study of 10 hospitals, the ability to adopt a culture that promoted performance improvement was linked to significant reductions in risk-standardized mortality rates.^21^ Moreover, the guiding coalitions (ie, quality improvement teams) in the six hospitals that were successful were distinguished in three ways, which highlight the manifestations of inclusive leadership that successfully capitalized on and navigated through diverse perspectives, professional identities, and experiences within hospital teams: (1) including staff from difference disciplines and levels in the organizational hierarchy, (2) encouraging authentic participation by the members, and (3) using distinct patterns of managing conflict (ie, having clear role definitions, working to surface minority viewpoints, and collectively revisiting the shared goal of saving lives).^22^ Based on this literature, adequately engaging diversity of viewpoints, experience, and staff roles can have a marked impact on health outcomes.

## Last, the Sense of Urgency

Finally, it is time to talk about action. Attracting diverse people (diverse in perspectives, professional identities, and experiences) to the work, engaging the pluralism of ideas and perspectives to unearth new ways of seeing old problems, and channeling the inevitable conflict into creative problem solving takes strong leadership and commitment at all levels. Nevertheless, these actions are evidence-based; the *way* we work together affects clinical experience and patient outcomes. Although the studies reviewed do not examine racial/ethnic diversity per se, they do lend insight into effectively navigating environments with extensive diversity of perspectives, professional identities, and experiences. Future research may assess whether these insights have application to other forms of diversity as well. In this time of extreme polarization and division globally and locally, health policy and management research has an opportunity to share evidence that could help navigate an increasingly diverse environment, at least within the field of healthcare, towards a more inclusive, humane, and life-giving approach to our collective future.

## Ethical issues

Not applicable.

## Competing interests

Author declares that she has no competing interests.

## Author’s contribution

EHB is the single author of the paper.
